# The entropic view of aging: from thermodynamics to biology

**DOI:** 10.1093/lifemedi/lnag020

**Published:** 2026-05-26

**Authors:** Zeming Wu, Shuai Ma, Si Wang, Moshi Song, Weiqi Zhang, Jing Qu, Juan Carlos Izpisua Belmonte, Guang-Hui Liu

**Affiliations:** School of Life Sciences, Jilin University, Changchun 130012, China; Aging Biomarker Consortium (ABC), Beijing 100101, China; Aging Biomarker Consortium (ABC), Beijing 100101, China; State Key Laboratory of Organ Regeneration and Reconstruction, Human Organ Physiopathology Emulation System, Institute of Zoology, Chinese Academy of Sciences, Beijing 100101, China; Beijing Institute for Stem Cell and Regenerative Medicine, Beijing 100101, China; University of Chinese Academy of Sciences, Beijing 100049, China; Aging Biomarker Consortium (ABC), Beijing 100101, China; Advanced Innovation Center for Human Brain Protection, National Clinical Research Center for Geriatric Disorders, Aging Translational Medicine Center, Beijing Municipal Geriatric Medical Research Center, Beijing Key Laboratory of Environment and Aging, Xuanwu Hospital Capital Medical University, Beijing 100053, China; Aging Biomarker Consortium (ABC), Beijing 100101, China; State Key Laboratory of Organ Regeneration and Reconstruction, Human Organ Physiopathology Emulation System, Institute of Zoology, Chinese Academy of Sciences, Beijing 100101, China; Beijing Institute for Stem Cell and Regenerative Medicine, Beijing 100101, China; University of Chinese Academy of Sciences, Beijing 100049, China; Aging Biomarker Consortium (ABC), Beijing 100101, China; University of Chinese Academy of Sciences, Beijing 100049, China; China National Center for Bioinformation and Beijing Institute of Genomics, Chinese Academy of Sciences, Beijing 100101, China; Aging Biomarker Consortium (ABC), Beijing 100101, China; State Key Laboratory of Organ Regeneration and Reconstruction, Human Organ Physiopathology Emulation System, Institute of Zoology, Chinese Academy of Sciences, Beijing 100101, China; Beijing Institute for Stem Cell and Regenerative Medicine, Beijing 100101, China; University of Chinese Academy of Sciences, Beijing 100049, China; Beijing Institute of Heart Lung and Blood Vessel Diseases, Beijing Anzhen Hospital, Capital Medical University, Beijing 100029, China; Altos Labs San Diego Institute of Science, San Diego, CA 94065, United States; Salk Institute for Biological Studies, La Jolla, CA 92037, United States; Aging Biomarker Consortium (ABC), Beijing 100101, China; State Key Laboratory of Organ Regeneration and Reconstruction, Human Organ Physiopathology Emulation System, Institute of Zoology, Chinese Academy of Sciences, Beijing 100101, China; Beijing Institute for Stem Cell and Regenerative Medicine, Beijing 100101, China; University of Chinese Academy of Sciences, Beijing 100049, China

## Abstract

A growing body of evidence suggests that aging is not simply the accumulation of damage, but a systemic progression of increasing entropy across biological scales. Synthesizing concepts from thermodynamics and information theory, we outline a tentative three-stage entropy model of the lifespan: order-building development, homeostatic adulthood, and disorder-dominant aging. Within this model, we attempt to reframe the established hallmarks of aging as interconnected nodes in an entropy-centered network and detail the multiscale manifestations of disorder from molecules to systems. To quantify this trajectory, we introduce a Multiscale Entropic Aging Index (MEAI) as a proof-of-concept conceptual framework, designed to integrate measurements of disorder across biological levels. This framework seeks to offer a potential unifying, quantitative language for aging research and suggests that entropy reduction could serve as a testable mechanism underlying interventions, laying a principled foundation for the future development of biomarkers and rejuvenative strategies.

## Introduction

Entropy, first defined in 1865 by Rudolf Clausius as a thermodynamic property, quantifies the disorder or randomness in a system macroscopically [1]. The second law of thermodynamics states that isolated systems naturally evolve toward higher entropy, a state of increased chaos and energy dissipation. In Clausius’s work, the core formula describes the change in entropy (*S*), not an absolute value. For a reversible process, the infinitesimal change in entropy is given by Equation (1):


(1) $$\text{d}S=\frac{\delta Q_{\text{rev}}}{T},$$

(d*S*, the infinitesimal change in the entropy of the system; δ*Q*_rev_, the infinitesimal amount of heat transferred to the system in a reversible process; *T*, the absolute temperature of the system at the moment of heat transfer)

In open biological systems, such as cells or organisms, entropy is continuously exchanged with the environment. Metabolic processes supply free energy that sustains cellular order, while irreversible dissipation generates entropy. A net increase in entropy reflects a state in which energy dissipation outweighs the system’s capacity for maintenance and repair, a condition commonly associated with aging. For example, mitochondrial dysfunction in aged cells reduces ATP production efficiency, potentially increasing entropy production at the subcellular level [2].

In 1877, Ludwig Boltzmann’s kinetic theory redefined it microscopically [3] by introducing Equation (2):


(2) $$S=k_{B}lnW,\;$$

(*k_B_*, Boltzmann’s constant; ln, the natural logarithm; *W*, the number of microstates corresponding to a macrostate).

*W* represents the number of distinct molecular/cellular configurations (microstates) of a biological system under a given phenotypic state (macrostate). For example, the 3D chromatin conformations of a young cell are restricted to a small set of functional microstates (low *W*, low *S*), while aged cells exhibit constitutive heterochromatin decondensation and thousands of aberrant chromatin conformations (high *W*, high *S*), directly increasing Boltzmann entropy at the epigenomic scale.

In information theory, Claude Shannon later adapted entropy to measure uncertainty in data, where higher entropy corresponds to greater unpredictability [4], as expressed by Equation (3):


(3) $$H(X)=-\sum_{i=1}^{n}p(x_{i})\mathrm{lo}g_{2}p(x_{i}),$$

(*H*(*X*), Shannon entropy of the random variable *X* (measuring the degree of disorder in the system); *n*, the total number of possible states or outcomes of *X*; *p*(*x_i_*), the probability of states or outcomes (e.g. methylated vs unmethylated status at a CpG site, or expression level of a gene) occurring).

Unlike thermodynamic entropy, Shannon entropy (i.e. information entropy) quantifies informational disorder in biological systems (e.g. DNA methylation states, gene expression profiles) [5, 6]. These concepts converge in biology: living systems resist entropy by harnessing energy to maintain order [7, 8], but aging represents the gradual failure of this resistance [9]. In this biological context, terms like “disorder” and “chaos” refer to the loss of functional and structural orderliness, which is the macroscopic manifestation of increasing entropy.

Biological order is epitomized during development and evolution, which organize matter into precise, low-entropy structures [10]. For example, embryonic development follows genetic and epigenetic blueprints to form specialized tissues, a process that is macroscopically entropy-reducing, with accumulating information content as cells differentiate into functionally distinct lineages. It is important to note that this progression toward organismal order can involve controlled stochasticity at molecular scales (e.g. in DNA methylation dynamics or chromatin remodeling [11]), which serves as essential “exploration” to reach the final low-entropy state. Evolution similarly optimizes traits for fitness, creating informational and functional complexity [12]. Conversely, aging is characterized by a systemic shift toward disorder across scales, reflecting the thermodynamic inevitability of entropy accumulation when the preservation of informational stability fails [13]. Here, we synthesize these contrasting dynamics to present aging through the lens of a lifelong entropy trajectory, suggesting that the accumulation of disorder might provide a unifying, quantifiable framework for understanding its mechanisms and guiding interventions.

## The three-stage entropy dynamics model over the lifespan

**Figure 1.**
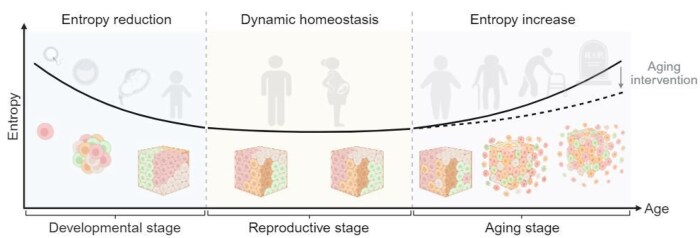
A three-stage model of entropy dynamics across the lifespan. A working model illustrating the three-stage entropy trajectory across the lifespan. The developmental stage is a period of entropy reduction, where biological complexity and information content increase as ordered cellular, tissue, and organism structures are established. The reproductive stage represents a state of dynamic homeostasis, where robust regulatory mechanisms (e.g. DNA repair, immune surveillance) maintain entropy at a stable, low level to support reproductive fitness. The aging stage reflects a phase of entropy increase, characterized by the breakdown of homeostatic mechanisms, leading to accumulating disorder. Rejuvenative interventions can counteract this aging-related entropy accumulation.

Building upon the framework of orderliness, we propose a hypothesis describing the lifelong trajectory of biological entropy, which unfolds in three distinct phases: programmed entropy reduction (developmental stage), dynamic homeostasis (adult reproductive stage), and systemic entropy increase (post-reproductive aging stage) (Fig. 1).

The first phase, programmed entropy reduction, dominates development. Starting from a single-cell zygote, organisms undergo division, differentiation, and morphogenesis to form complex compartmentalized, polarized structures (macroscale entropy reduction). This process involves biological reprogramming at multiple levels. Molecularly, for example, the establishment of precise DNA methylation and histone modification landscapes and the reorganization of chromatin into specific 3D architectures write biological information into the genome, ensuring correct gene expression patterns while defining cell fates [11, 14]. This is a quintessential information-accumulating, entropy-reducing process, where information is written and disorder is dispelled to create a highly ordered state [12].

The second phase represents a state approaching dynamic homeostasis of entropy production and dissipation throughout the reproductive adulthood. At this stage, robust maintenance mechanisms, such as DNA repair, protein turnover, and immune surveillance, counteract perturbations to sustain relatively stable, low entropy levels, supporting successful reproduction [13, 15–17]. Notably, even in adulthood, low-level damage accumulation occurs (e.g. oxidative DNA lesions), but these are cleared before they can drive systemic entropy elevation, thus representing a net entropy-balancing process.

Finally, the phase of systemic entropy increase characterizes post-reproductive aging. Once the core reproductive mission is accomplished, the individual’s contribution to the population gene pool declines, and the force of natural selection weakens. The substantial energetic and resource costs of maintaining a low-entropy, high-informational-integrity state no longer confer a clear evolutionary advantage. Consequently, maintenance systems falter [15–18], and unregulated entropy accumulation becomes dominant, distinguishing this from isolated damage, as entropy can integrate diverse damage types into a systemic collapse of order (e.g. DNA damage + epigenetic drift + proteostasis loss drive a net increase in cellular microstates, elevating Boltzmann entropy and disrupting cell identity). This ultimately links the loss of order to phenotypic hallmarks of aging [13].

This three-stage trajectory reconciles programmed and entropy theories of aging: the post-reproductive decline of somatic maintenance programs serves as a temporal permissive condition for entropy accumulation, while entropy dynamics mechanistically describe how disorder propagates across scales once this homeostatic capacity wanes. In this view, the program does not actively drive aging but sets the stage for thermodynamic inevitability. This model further suggests the possible existence of an entropic tipping point, a threshold beyond which molecular disorder may become self-amplifying, potentially contributing to the nonlinear rise in morbidity and mortality observed in human aging curves. Such a tipping point could reflect the macroscopic manifestation of cumulative microstate expansion across scales, shaped by both programmed maintenance robustness and stochastic entropic insults. The population-level aging inflection point might thus be reframed as an echo of an underlying entropic phase transition, offering a conceptual target for interventions aimed at postponing this critical threshold. It is critical to recognize that entropy changes may not synchronize across all biological scales simultaneously. For example, during aging, molecular-scale entropy (e.g. in the (epi)genome) might accumulate first, eventually triggering a sharp rise in cell-, tissue- and system-scale entropy, marking functional collapse.

## Entropy increase in aging: a multiscale perspective

### Molecular and subcellular disorder

**Figure 2.**
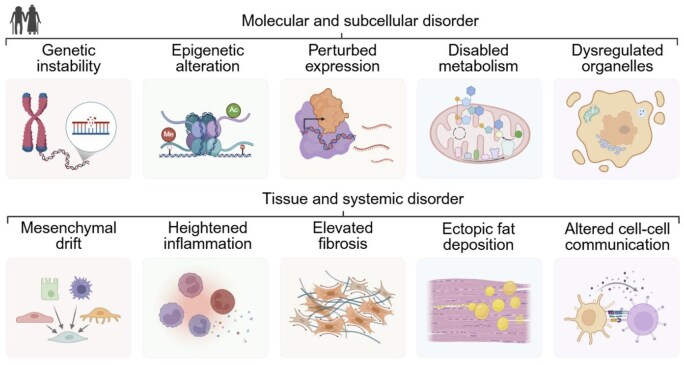
Multiscale manifestations of entropy increase during aging. Entropy increase across biological scales during aging. At the molecular and subcellular level, aging-related disorder includes genetic instability (e.g. DNA damage and mutations), epigenetic alteration (e.g. DNA methylation drift), perturbed expression (dysregulated transcriptomic and proteomic profiles), disabled metabolism (dysfunctional metabolic signaling), and dysregulated organelles (morphological and functional defects in subcellular compartments). At the tissue and systemic level, aging-related disorder encompasses mesenchymal drift (the shift of non‑mesenchymal cells or tissues toward mesenchymal characteristics, indicating an erosion of original identity), heightened inflammation (chronic, unresolved immune activation), elevated fibrosis (excessive extracellular matrix deposition and fibroblast infiltration), ectopic fat deposition (abnormal lipid accumulation in non-adipose tissues), and altered cell–cell communication (disrupted intercellular signaling networks). Together, these representative changes reflect the loss of structural and functional order from molecular to tissue and organism levels, which aligns with the entropic framework of aging—where rejuvenative interventions counteract this trend by restoring low-entropy states.

Aging drives a systemic increase in entropy across all biological scales (Fig. 2). Genomically, rising informational entropy parallels genomic instability, which encompasses DNA damage, mutations, telomere attrition, and retrotransposon reactivation, contributing to age-related diseases such as neurodegenerative disorders [19–24]. Epigenetically, entropy increases through altered DNA methylation, histone modification redistribution, and chromatin remodeling, leading to loss of compartmentalization and gene dysregulation [5,20,25–32]. For example, in Chinese individuals, epigenetic entropy, which is calculated as the Shannon entropy of CpG methylation states, shows a significant increase with age when comparing elder (> 70 years old) to younger adults (45–70 years old); whereas, long-lived individuals (> 90 years old) display a lower methylation entropy than elderly controls, which correlates with preserved heterochromatin structure and reduced age-related disease risk [28].

Transcriptionally, the leaky expression of developmentally restricted genes (e.g. placenta-specific genes (*PSG*s), *PAPPA*) and repetitive elements (e.g. endogenous retroviruses (*ERV*s)) in senescent cells represents a manifestation of elevated transcriptional entropy [26,30,31,33–35]. The aging-driven disruption of gene expression pattern at the transcriptional level amplifies transcriptional noise or expression divergence, underscoring the loss of inherent transcriptional order [36–39]. Recently, a concept of network structural entropy derived from single-cell RNA sequencing data has been proposed. Studies applying this metric have demonstrated its increase in senescent cells, which correlates with the dysregulation of inherent cellular functions [40]. Moreover, single-cell transcriptome-based measures of cellular entropy not only correlate with senescence but also exhibit oncogenic potential, thereby linking transcriptional disorder to both proliferative arrest and cancer risk [41]. Yet, existing supervised approaches for detecting cellular senescence from single-cell transcriptomic data, while capable of generating cell-type-specific senescence scores, are limited by their dependence on annotated data and lack generalizability. A proposed universal framework overcomes this by quantifying single-cell transcriptional entropy through deviation from the transcriptomic manifold, capturing the breakdown of transcriptional coordination. This method identifies aging-affected cell types and distinguishes loss of expression precision from activated stress responses in high-entropy cells [42], offering a novel perspective for applying transcriptional entropy in aging assessment.

At the protein level, misfolding and aggregation, such as Aβ deposition observed in aged neuronal cells and associated with Alzheimer’s disease, lead to unregulated diversity of protein conformations and loss of functional specificity [43]. From a systemic perspective, this can be quantified as a reduction in the orderliness of the proteostatic network, or an increase in “proteostatic entropy”. Similarly, perturbations in nutrient sensing and metabolism disrupt the coordination and stability of metabolic networks, characterized by the dysregulation of key metabolic regulators, metabolites, enzymes, and even ions [43, 44]. For example, the recently defined ferro-aging pathway, driven by iron overload and lipid peroxidation, serves as a compelling molecular instantiation of the entropic principle, wherein stochastic oxidative damage to macromolecules manifests as a progressive, system-wide increase in disorder and functional decline [45]. This process reflects a decrease in the orderliness of metabolic systems, which can be conceptualized as an elevation of “metabolic entropy”. These molecular disorders together reflect the loss of informational and functional integrity within the cell.

Subcellular structures also lose their ordered architecture with aging (Fig. 2). For example, aged cells typically exhibit ruptured nuclear envelope and irregular nuclear morphology, as well as structural dysregulation in nuclear pores and nucleoli [43, 46–50]. Beyond the increasing chaos in the nucleus, a variety of organelles in the cytoplasm also display a rise in structural entropy. For example, the number of swollen or cristae-disorganized mitochondria often increases with aging of the cell [43, 51, 52]. Similarly, disrupted endoplasmic reticulum arrangement and abnormal lysosomal size and number also serve as potential indicators of increased structural entropy in organelles [20, 43, 53, 54]. Such structural disorder correlates with functional decline and loss of cellular homeostasis, underscoring the multiscale nature of entropic accumulation in aging.

### Tissue and systemic disorder

Tissue-level entropy manifests as the loss of spatial order and functional specialization, suggesting escalating chaos in physiological, histological, cellular, and molecular features with aging (Fig. 2). For example, a large cohort study based on magnetic resonance imaging analysis reflecting brain activities revealed an increase in functional entropy with aging [55–57]. An entropy model based on electrocardiogram data has been reported to predict aging-related outcomes including fracture and mortality risks, which not only reflects physiological disorder at the organ level (i.e. heart) but also serves as a systemic indicator of homeostatic dysregulation at the organismal level [58]. Single-cell RNA sequencing of aging human lung tissues demonstrated a concurrent increase in somatic mutations, transcriptional perturbations, and shifts in cellular composition [59]. This convergence reveals a loss of inherent order across multiple levels and a corresponding rise in transcriptional entropy. A recent work introduced the concept of organizational structure entropy (OSE) for measuring the degree of disorder in tissue aging using spatial transcriptomic data and identified an increased OSE in aged tissues like hippocampus, spleen, and liver. Moreover, tissues with high OSE values preferentially show an enrichment of immune cells and a loss of cellular identity [60].

Consistent with the loss of identity, a gene expression profiling study across more than 40 tissue types revealed that aging is associated with a broad downregulation of pathways maintaining core cellular functions and a concurrent upregulation of genes related to epithelial–mesenchymal transition [61]. This phenomenon has been termed “mesenchymal drift”, which extends beyond epithelium to encompass endothelial cells, pericytes, and macrophages, leading to a collective “identity blurring”—essentially loss of cell type-specific informational identity [61]. These cells do not fully transdifferentiate but acquire superimposed mesenchymal traits, impairing their original functions while endowing them with new capabilities, such as modifying the extracellular matrix and altering tissue stiffness. Importantly, the pervasive mesenchymal drift signature is implicated in the pathogenesis of multiple age-related chronic diseases, including idiopathic pulmonary fibrosis, metabolic dysfunction-associated steatohepatitis, and Alzheimer’s disease [61]. Such drift can be viewed as a manifestation of informational loss (or an increase in entropy) and may constitute a fundamental mechanism driving organ dysfunction, aging, and chronic disease progression [62].

Apart from the infiltration of immune cells, aged tissues commonly exhibit an upregulation of inflammatory factors, a state referred to as the senescence-associated secretory phenotype (SASP), demonstrating pan-tissue inflammation during aging [43]. Such disorder is further manifested by structural alterations such as fibrosis and ectopic fat accumulation. Fibrosis, driven by fibroblast expansion and high extracellular matrix expression, disrupts tissue order in organs like heart, liver, and lungs. Similarly, ectopic fat deposition in non-adipose organs (e.g. skeletal muscle, liver, bone marrow) compromises tissue identity and function [43]. The breakdown of orderliness is further compounded by a decline in physiologically appropriate intercellular interactions. For instance, disruption of intercellular tight junctions, as recently reported in the aged primate small intestine, impairs epithelial barrier integrity and contributes to tissue disorganization [63]. Moreover, noise and chaos in cell-to-cell communication emerge as non-specific, destructive signals (e.g. the aforementioned pro-inflammatory signals) are indiscriminately released, disrupting the signaling balance of the local microenvironment [64]. For example, recent studies have uncovered that aged tissues accumulate immunoglobulins, especially IgG, which drives a senescence-associated phenotype and fuels multi‑organ inflammation [60,65]. At a finer scale, cellular senescence within aged tissues introduces multidimensional molecular disturbances across the genome, epigenome, transcriptome, proteome, and metabolome, amplifying tissue-level disorder [16,43,60,66–68].

Systemically, as beneficial hormonal cues wane and disruptive signaling emerges, the integrative capacity of vital systems, including the nervous, endocrine, and immune systems, is impaired, leading to decreased homeostasis and increased vulnerability to age-related diseases [69]. This shift from stability to disorder indicates a rise in organismal entropy, though quantitative models are still needed to formally establish this link. The most direct physiological manifestation of this transition is a marked decline in organismal resilience and a corresponding increase in fragility [70–77]. Here, “resilience” refers to the ability of a system to restore its original steady state after a disturbance (such as infection, trauma, or thermal stress), while “fragility” denotes the tendency of a system to undergo functional collapse under perturbation. During aging, multi-scale increases in entropy, from molecular to cellular, tissue, and systemic levels, progressively erode the redundancy and coordination necessary for sustaining complex functions. Consequently, when confronted with stressors, the aged organism not only recovers more slowly (loss of resilience) but is also more likely to cross a critical threshold into irreversible functional decline or disease (increased fragility). This reflects the approach of the system as a whole toward a critical threshold of disorder, providing an entropy-based dynamical explanation for the steep rise in age-related morbidity and mortality. This systemic loss of homeostatic order has been empirically captured in a human cohort, where a rising dyshomeostasis index (DHI), which integrates multi-omics and clinical parameters, quantitatively reflects the entropic principle of increasing disorder across multiple physiological systems [78].

## Entropy as a unifying framework for aging research

### How the entropic framework refines and unifies the hallmarks of aging

The entropic framework is more than a relabeling of known hallmarks; it provides a unifying, quantitative lens that refines our understanding. The current hallmarks framework [79–81], while highly influential, catalogs an expanding list of features but leaves their interrelationships largely unspecified, nor does it identify which might operate most upstream. The entropy framework offers a complementary perspective: it posits that regardless of the initial insult, if it ultimately increases the number of accessible system microstates and degrades informational capacity, it contributes to aging. Conversely, if a given type of damage does not entail such entropic increase, it may not represent a core driver of the aging process, a distinction that is, in principle, empirically testable. It further suggests that diverse age-related changes are interconnected manifestations of a single thermodynamic trend, i.e. loss of structural and functional information and order. This perspective shifts the focus from cataloging independent types of damage to investigating how entropy propagates and amplifies across scales, potentially leading to systemic failure at critical thresholds. The core value of the entropic perspective lies in its ability to recast established aging hallmarks as interconnected nodes in a network driving systemic disorder, prompting novel, quantifiable questions. Importantly, it generates the testable prediction that effective aging interventions should produce a measurable decrease in entropy metrics at one or more scales, providing a universal benchmark for efficacy beyond specific physiological outcomes.

### Assessment: quantitative entropy-based biomarkers

Biological entropy is commonly quantified using Shannon’s formula. This measure facilitates cross-scale disorder comparison from molecular states to system-level function. Critically, it allows different types of disorder (e.g. epigenetic drift, transcriptional perturbation) to be placed on a common quantitative scale. Increased entropy at genomic, epigenetic, transcriptional, and functional levels has been demonstrated using this approach, showing predictive potential for cellular and tissue aging that can extend beyond conventional metrics [5, 19, 26, 41, 55, 60, 82]. For instance, methylation entropy has been shown to be lower in healthy long-lived individuals and can distinguish health status independently of chronological age [28]. Similarly, transcriptional entropy calculated from single-cell RNA-seq data can identify pre-malignant cell states not captured by traditional markers [41].

While individual entropy metrics offer powerful biomarkers, translating the entropic theory of aging into a unified, practical index faces two critical challenges. First, the non-monotonicity of entropy change over the lifespan presents a conceptual hurdle: if the central hypothesis holds, that entropy decreases during development, reaches a nadir in young adulthood, and increases thereafter, then a given entropy value could correspond to two distinct biological ages (one in development, one in aging), making it an ambiguous standalone measure of aging. Second, there is the multiscale complexity of biological systems. Aging is a systemic, heterogeneous, and nonlinear process manifesting across distinct dimensions (molecular, cellular, tissue, etc.) [16, 83, 84], each requiring specific quantification methods. A valuable aging index must therefore integrate this complexity into a coherent single metric.

To explore a potential solution to these limitations, we here propose a conceptual framework to measure entropy across multiple biological scales, from molecules to tissues, and combine them into a hypothetical composite index. This approach circumvents the non-monotonicity problem in theory by using the young adult homeostatic state as a reference point, focusing on the entropic increase that characterizes the aging phase. This leads to the formulation of a Multiscale Entropic Aging Index (MEAI) as a theoretical construct, given by Equation (4):


(4) $$\mathrm{MEAI}=\sum_{i=1}^{M}w_{i}\frac{H_{i}^{\left(\mathrm{current}\right)}-\mu_{H_{i}^{\left(\mathrm{ref}\right)}}}{\sigma_{H_{i}^{\left(\mathrm{ref}\right)}}},$$

Here, *M* represents the number of biological scales selected. The weight *w_i_* (where ∑*w_i_* = 1) reflects the relative contribution of each scale’s disorder to systemic aging. *H_i_*^(current)^ represents the entropy (e.g. genetic entropy, epigenetic entropy, transcriptional entropy, proteomic entropy, metabolic entropy, cell type entropy, tissue organization entropy, functional entropy) at biological scale *i* for the individual being assessed. *μ_H_i__*^(ref)^ represents the mean entropy at biological scale *i* in a large, well-characterized young healthy reference population (where entropy reaches a stable nadir post-development). *σ_H_i__*^(ref)^ represents the standard deviation of entropy at biological scale *i* in the reference population. Notably, since MEAI is designed to specifically quantify aging-related entropic increase, the formula holds under the premise that *H_i_*^(current)^ > *μ_H_i__*^(ref)^. The MEAI offers a theoretical basis for quantifying relative entropic increase across scales, representing a hypothetical single metric that would, in principle, increase with accumulated disorder, though its practical implementation as a unified index remains pending.

As a proof-of-concept, MEAI requires extensive implementation, optimization, and empirical validation to address unmet practical challenges. First, the assignment of *w_i_* is non-trivial and may be informed by data-driven approaches (e.g. feature importance from machine learning models) or expert consensus, yet it must ultimately be refined through testing on large-scale longitudinal datasets. Second, ensuring comparability of entropy values across disparate biological scales necessitates a consistent computational framework (e.g. standardized state/bin definitions for Shannon entropy calculation). Third, defining the robust reference values (*μ_H_i__*^(ref)^ and *σ_H_i__*^(ref)^) is critical. This requires establishing a baseline from a clearly characterized cohort of healthy young individuals, with mean/median entropy from large cohorts (strict health criteria) and stratified values (e.g. by sex) to boost population applicability. Fourth, linear weighted summation may oversimplify nonlinear synergies between multi-scale disorder, advanced models (e.g. graph neural networks, multi-scale factor analysis) may be needed to capture how molecular entropy amplifies tissue and systemic dysfunction. Fifth, computational feasibility remains a hurdle: some entropy metrics (e.g. tissue structural entropy from spatial transcriptomics, organ functional entropy from imaging) lack standardized pipelines, requiring reproducible tool development for cross-scale quantification. Given its current theoretical nature and unresolved practical challenges (e.g. cross-scale entropy comparability, weight calibration, individual-level stability, and nonlinear integration), the predictive performance of MEAI and its optimization in diverse populations remain to be explored and established through large-scale future empirical studies. Initial proof-of-concept studies for MEAI should prioritize integrating two to three well-validated entropy metrics (e.g. DNA methylation entropy + single-cell transcriptional entropy), testing whether the composite index outperforms single metrics in predicting aging phenotypes or intervention efficacy, this incremental validation will refine the framework before expanding to complex multi-scale integration.

Notably, MEAI’s reliance on Shannon entropy introduces a fundamental methodological consideration. As a distribution-based metric, Shannon entropy requires a predefined probability distribution across discrete states or bins, an inherent characteristic that complicates assigning meaningful entropy values to individual samples without reference to a population baseline. This limitation underscores the broader challenge of personalized aging assessment, where single-individual measurements need to be interpreted against normative data. In light of these considerations, recent methodological discussions have highlighted complementary approaches such as the Mahalanobis distance, which quantifies how far an individual’s multi-dimensional biomarker profile deviates from a healthy reference population [58, 85]. Unlike Shannon entropy, this metric can be calculated from a single observation across multiple variables and may offer a more direct measure of physiological divergence, one that could complement the cross-scale entropy integration envisioned by MEAI, once its practical limitations are addressed in future work.

Collectively, translating the entropic aging framework and realizing the potential of MEAI as a unified index from theory to practice hinges on several critical, yet unmet steps: building standardized population cohorts to capture normative aging trajectories, standardizing entropy calculations across scales, establishing robust reference values, and developing integrated pipelines to synthesize multi‑scale measures into a coherent “entropic age” index. Future advances will likely rely on machine‑learning models trained on large multi‑omics cohorts, potentially hybridizing entropy‑based and distance‑based measures to capture both systemic disorder and individual physiological divergence, thereby paving the way for a next generation of mechanism‑informed biomarkers.

### Intervention: targeting entropy reduction with mechanistic specificity

Rejuvenative interventions that target aging-related biological processes leverage natural “negative entropy”-associated mechanisms to restore functional and structural order (Fig. 1). This framework allows us to contextualize their actions in entropic terms: interventions improve core biological functions, with reduced systemic disorder (entropy) emerging as a downstream readout of these beneficial changes. For example, caloric restriction may lower systemic entropy by enhancing metabolic efficiency and selective autophagy, specifically removing high-entropy, dysfunctional cellular components like damaged mitochondria [86–88]. Exercise, while transiently boosting energy expenditure, drives long-term improvements in biological order by preventing DNA damage and telomere shortening, mitigating DNA methylation drift, and restoring heterochromatin stability [9, 89, 90]; such enhancements in genomic and epigenetic integrity potentially reflect the reduced genomic and epigenetic entropy.

Recent studies have demonstrated that inactivation of a single factor (e.g. SIRT2, SIRT5, and GRHL2) or activation of others (e.g. KAT7, ERVs, and LINE-1) is sufficient to initiate the programmed aging process, while targeted intervention of these factors can achieve rejuvenation through reprogramming by resetting core transcriptional programs and restoring information homeostasis and order [34, 91–95]. This suggests that maintaining the expression control capacity of individual factors enables the system to preserve its overall negentropic capacity. Similarly, rejuvenation strategies, including administering geroprotective compounds (e.g. metformin, vitamin C, and uridine) or delivering youth-promoting genes (e.g. *CLOCK*, *SOX5*, and Yamanaka factors), can counteract aging by reestablishing genetic and epigenetic order [96–107]. Such restoration of molecular organization aligns with the conceptual framework of reduced biological entropy as a downstream outcome. Furthermore, the strategy of intermittently introducing Yamanaka genes has been shown to alleviate aging phenotypes through reversing mesenchymal drift, which points to the re-establishment of cellular identity order [61]. Other interventions such as senolytics directly remove high-entropy senescent cells, a major source of disorder in aged tissues, while stem cell transplantation introduces low-entropy, information-rich elements to reset local tissue organization [108–110]. These actions improve tissue structure and function, with reduced multi-scale entropy serving as a unifying readout of their biological efficacy. Therefore, a central, testable prediction of this framework is that diverse aging interventions, by improving distinct biological mechanisms, should converge on reducing measurable entropy at multiple scales, offering a potential universal biomarker for their deep biological effect.

## Conclusion and future perspective

In summary, aging can be usefully viewed as a systemic process of entropic accumulation, spanning molecular disorder, cellular identity loss, and tissue dysfunction. As a working hypothesis, this framework unifies aging hallmarks by explaining them as interconnected contributors to a common trend toward rising disorder, with entropy metrics emerging as versatile biomarkers, ranging from (epi)genomic and transcriptional entropy for predicting cellular senescence, to entropy relying on cellular identity loss for quantifying tissue degeneration, and further to imaging-derived brain entropy for assessing aging and Alzheimer’s disease, as well as electrocardiogram-based entropy for forecasting aging-related adverse outcomes. Moving beyond framing entropy as primarily a metaphorical descriptor of aging, this framework seeks to advance a paradigm shift from philosophical analogy toward a rigorous operational scientific construct: by establishing multiscale operational definitions of biological entropy, from epigenetic methylation entropy to tissue structural entropy, and introducing MEAI as a relative metric that circumvents the non-monotonicity problem, it proposes to define entropy as a quantifiable state variable that could potentially unify programmed and stochastic views of aging within a common conceptual coordinate system.

This entropy-based view is further reinforced by its convergence with the Information Theory of Aging (ITOA), which posits the loss of epigenetic information as a molecular driver of aging [111]. The two perspectives are mutually illuminating: ITOA provides a mechanistic basis for “molecular entropy increase”, wherein noise-induced epigenetic disorder fuels cellular identity loss and expression chaos, mirroring multi-scale entropic increase. Both frame aging as systemic disorder amplification, from molecular to systemic function, and align on a dynamic trajectory across the lifespan: development (epigenetic writing/entropy decrease), adulthood (information homeostasis/entropy balance), and aging (information loss/entropy increase). Critically, ITOA translates the goal of “reducing disorder” into actionable strategies like partial reprogramming and enhanced DNA repair, aiming to lower molecular entropy by restoring youthful epigenetic landscapes. While ITOA focuses on epigenetic information as a key substrate, the present framework attempts to generalize “information” to encompass order across multiple biological scales, from epigenetic to transcriptional, proteostatic, metabolic, and tissue structural entropy. In this view, epigenetic entropy is but one manifestation of a broader systemic trend; the framework thus may help address how disorder propagates from molecules to organismal function, offering a complementary systems-level perspective that is consistent with ITOA while potentially extending its conceptual reach.

At present, no empirical evidence supports the linear additivity of entropy across scales; thus MEAI remains a conceptual scaffold rather than a ready‑to‑use metric. Looking forward, quantifying entropy via conceptual frameworks like MEAI, anchored to young adult reference entropy and requiring future calibration of data-driven weights, complemented by Mahalanobis distance for personalized assessment, offers potential for yielding cross-scale aging biomarkers with unique integrative potential in the future, while entropy-reversing interventions hold great promise for extending healthspan. Realizing this vision requires a concerted effort to develop standardized, cross-scale bio-entropy protocols, build large-scale “entropic phenotype” databases, and validate such framework in large longitudinal studies to address its current practical limitations. A speculative hypothesis worth testing is whether organ functional collapse might consistently follow a simultaneous, sharp rise in entropy across multiple scales (an “entropic critical point”). Interdisciplinary collaboration spanning thermodynamics, omics, clinical medicine, and artificial intelligence is essential to decode aging’s “entropic signature” and transition from managing late-life diseases to targeting the root disorder of the aging process itself.
